# Interdisciplinary collaboration and non-pharmacological interventions to improve mental health of older adults in residential care facilities (part 2)

**DOI:** 10.1192/j.eurpsy.2026.10277

**Published:** 2026-07-31

**Authors:** K. Gillis, D. L Gerritsen

**Affiliations:** 1 Centre for Research and Innovation in Care, Universiteit Antwerpen, Antwerpen, Belgium; 2 Primary and Community Care, Radboud University Medical Center, Nijmegen, Netherlands

## Abstract

**Abstract:**

The presentation will go further into needs-based interventions representing interdisciplinary collaboration, and how an academic knowledge network of a University Hospital and 18 long term care organizations provides the context for developing, evaluating and implementing these. It will elaborate on the Act in case of Depression intervention, a cyclic intervention developed for detecting and treating depression in nursing homes. This intervention was tested in a stepped-wedge cluster randomized trial which showed that it increased Quality of life in nursing home residents, lowered depression prevalence in residents living in nursing home units for somatically frail residents and lowered apathy in residents of specialized dementia units. Further, the development of the Shared Action for Breaking through Apathy intervention for people with dementia in nursing homes will be illustrated.

**Disclosure of Interest:**

None Declared

